# Genome-Wide Identification, Evolutionary Analysis and Expression Profiles of *LATERAL ORGAN BOUNDARIES DOMAIN* Gene Family in *Lotus japonicus* and *Medicago truncatula*

**DOI:** 10.1371/journal.pone.0161901

**Published:** 2016-08-25

**Authors:** Tianquan Yang, Genwang yue Fang, Hua He, Jianghua Chen

**Affiliations:** 1 Key Laboratory of Tropical Plant Resources and Sustainable Use, Xishuangbanna Tropical Botanical Garden, Chinese Academy of Science, Kunming 650204, Yunnan Province, China; 2 University of Chinese Academy of Science, Beijing 100049, China; Wuhan University, CHINA

## Abstract

The *L**ATERAL ORGAN* *B**OUNDARIES* *D**OMAIN* (*LBD*) gene family has been well-studied in *Arabidopsis* and play crucial roles in the diverse growth and development processes including establishment and maintenance of boundary of developmental lateral organs. In this study we identified and characterized 38 *LBD* genes in *Lotus japonicus* (*LjLBD*) and 57 *LBD* genes in *Medicago truncatula* (*MtLBD*), both of which are model legume plants that have some specific development features absent in *Arabidopsis*. The phylogenetic relationships, their locations in the genome, genes structure and conserved motifs were examined. The results revealed that all *LjLBD* and *MtLBD* genes could be distinctly divided into two classes: Class I and II. The evolutionary analysis showed that Type I functional divergence with some significantly site-specific shifts may be the main force for the divergence between Class I and Class II. In addition, the expression patterns of *LjLBD* genes uncovered the diverse functions in plant development. Interestingly, we found that two LjLBD proteins that were highly expressed during compound leaf and pulvinus development, can interact via yeast two-hybrid assays. Taken together, our findings provide an evolutionary and genetic foundation in further understanding the molecular basis of *LBD* gene family in general, specifically in *L*. *japonicus* and *M*. *truncatula*.

## Introduction

LATERAL ORGAN BOUNDARIES DOMAIN (LBD) proteins, a plant-specific transcription factor family, possess a characteristic N-terminal LOB domain and play important roles in many aspects of plant development. The LBD protein typically contains four highly conserved cysteine (C) residues in a CX_2_CX_6_CX_3_C zinc finger-like motif (also called C block, where X represents variable residues) that is suggested to play crucial role in DNA binding. In addition, two other conserved features are found in the N-terminal half of the LBD: an invariant glycine residue and a leucine-zipper-like sequence LX_6_LX_3_LX_6_L [1,2]. Usually, the *LBD* gene family can be divided into two classes (class I and II) based on conserved motif number and structural features. Typically, class I members contain a CX_2_CX_6_CX_3_C motif and a leucine-zipper-like motif, while class II members contain only a CX_2_CX_6_CX_3_C motif. As transcription factors, LBD proteins function in the nucleus and bind to the conserved nucleotide consensus sequence GCGGCG. In addition, there is evidence that the interaction between LBD and bHLH proteins can reduce the DNA-binding affinity of LBDs [3].

Studies show that *LBD* genes usually exhibit temporal or tissue-specific expression patterns [1]. For example *LBD* genes are expressed in specialized regions such as the adaxial base of lateral organs, shoot apical meristem and boundary between lateral organs, indicating their important function in plant lateral organ development [4,5]. In *Arabidopsis*, *LBD* genes are found to involve in various tissues development such as leaf development [6] and lateral root initiation [7–9], as well as signaling transduction such as cytokinin [10] and gibberellin pathways [11]. Also, three *LBD* genes in *Arabidopsis* including *AtLBD37*, *AtLBD38* and *AtLBD39*, are implicated in anthocyanin biosynthesis and nitrate metabolism [12]. In other species, a panel of *LBD* genes were also well studied and characterized. For instance, the maize *Ramosa2* gene plays a role in regulation of inflorescence architecture [13]. Two maize LBD family genes RTCS and RTCL cooperatively act in shoot-borne root formation [14]. The rice auxin-inducible *ARL1* gene encodes a LBD protein that promotes adventitious root formation [15].

With the advent of high-throughput sequencing techniques, genome-wide identification and characterization of *LBD* genes have been conducted in *Arabidopsis* [1], rice [16], maize [17] and apple [18]. However, little is known about *LBD* genes in legumes to date. Legumes are important crop plants that not only have the unique ability to fix nitrogen from the atmosphere, but also are rich sources of protein and oil for the human diet. The genomes of many legume plants have recently been sequenced, providing an opportunity for detailed characterization of *LBD* genes in this important family of plants. Among legumes, *Lotus japonicus* and *Medicago truncatula* are used as model legumes due to their short life cycles, self-fertility, and relatively small diploid genome. In particular, these two species provide an excellent system for study of some specific development features including compound leaf development, motor organ specification, and root nodule formation that are absent in *Arabidopsis*. For example, *ELP1 (Elongated Petiolule1*; also called *MtLBD13* in this study) and *SLP* (*Sleepless*, also called *LjLBD6* in this study) were isolated from *M*. *truncatula* and *L*. *japonicus*, respectively, and found that they are involved in specification of so-called motor organ or pulvinus identity, uncovering a novel function for *LBD* genes in the determination of motor organs and the control of plant movement in legumes [5]. In addition, three *LBD* genes (*LjLOB1*, *LjLOB3* and *LjLOB4*) have been isolated from *L*. *japonicus*, and their specific expression patterns strongly showed that *LjLOB1* and *LjLOB3* (*al*so called *LjLBD11* and *LjLBD6* in this study) might have important roles in determining compound leaf development, and that another gene *LjLOB4* (*al*so called *LjLBD22* in this study) may be involved in floral development [4]. Thus, investigation of *LBD* genes at genome-wide level in legumes would likely provide new insights into the regulatory mechanisms of plant growth and development, especially, motor organs and compound leaves development.

In the present study, we identified and characterized the *LBD* gene family in *L*. *japonicus* and *M*. *truncatula* on the genome-wide scale. In total, we found 38 putative *LBD* genes in *L*. *japonicus* and 57 members in *M*. *truncatula*, respectively. Their motif distribution, evolutionary relationship and expression profiles were further characterized in detail. Importantly, we found that two LBD members *LjLBD11* (*LjLOB1*) and *LjLBD6* (*LjLOB3*, *SLP1*) were highly expressed in compound leaf and can form complex by proteins interaction, implying that they may work together in controlling compound leaf development in *L*. *japonicus*. These results obtained from this study provide global information important for further understanding the molecular functions of the *LBD* gene family in legumes.

## Materials and Methods

### Screening of *LBD* gene members

All *Arabidopsis* LBD proteins were retrieved from the DATF database (http://datf.cbi.pku.edu.cn) based on the previous study [1]. All protein sequences of *L*. *japonicus* and *M*. *truncatula* were downloaded from the database (http://www.plantgdb.org/LjGDB/) and Phytozome database (https://phytozome.jgi.doe.gov/pz/portal.html#!info?alias=Org_Mtruncatula), respectively. Using BLASTP program, all *Arabidopsis* LBD protein sequences were used as query against corresponding protein database. The hits with a significant E-value (< lE-3) and more than 70% identity were collected and considered as candidate proteins. Subsequently, redundant sequences or incomplete ORF sequences were removed from our gene set. Finally, all candidate proteins were further subjected to SMART (http://smart.embl-heidelberg.de/) and Pfam (http://www.pfam.sanger.ac.uk/) to confirm the presence of LOB domain.

### Chromosomal localization and gene structure analysis

To determine the distribution of LBD genes on chromosomes, position information of each *LBD* gene was obtained from genome annotation file (downloaded from website: http://www.plantgdb.org/LjGDB/ and http://www.jcvi.org/medicago/, respectively). A custom MATLAB script was used to draw the location information of LBD genes in chromosome or scaffold. According to the alignment between full-length CDS and corresponding gene sequence, exon-intron organization for individual *LBD* gene was illustrated by using Gene structure display server software (GSDS, http://gsds.cbi.pku.edu.cn/)

### Phylogenetic tree, evolutionary analysis and conserved motifs

Multiple sequence alignment of amino acid sequences was carried out using Clustalx v2.1 (http://www.clustal.org/clustal2/), and phylogenetic trees were then generated using the neighbor-joining (NJ) method with 10,000 bootstrap replicates in MEGA5.0 [19]. The functional divergence for classes or subclasses was estimated using the DIVERGE v3.0 package [20]. The coefficients of type-I (θ_I_) and type-II (θ_II_) functional divergence between any two classes were estimated based on the ML algorithm. The Type-I functional divergence means that some significantly site-specific changes mainly occurs between two classes. The Type-II means that functional divergence between two classes were mainly due to some site-specific shifts in amino acid properties. The coefficients of Type I or type II functional divergence with greater than zero were considered significant. Additionally, to identify which sites are critical for functional divergence between two classes, a site-specific posterior probability were predicted with a cut-off value more than 0.9 to reduce possible false positives.

Besides, conserved motifs of each LBD protein were identified using MEME Suite (http://meme-suite.org/) with parameters set: optimum width 6–200 amino acids and maximum number of motifs 15, and then visualized by MAST (Motif Alignment and Search Tool). In addition, the molecular weight (MW, Da) and isoelectric point (pI) of each protein were estimated by online ExPASy programs (http://www.expasy.org/tools/).

### Expression profiles of *LBD* genes in *L*. *japonicus*

The expression profiles of all *LjLBD* genes were investigated among different tissues or developmental stages using *L*. *japonicus* gene expression altas (LjGEA, http://ljgea.noble.org/v2/) [21]. Briefly, probe ID of each *LjLBD* gene was firstly obtained by gene sequence BLASTN program that is available at LjGEA website. If a given gene has more than one probe ID, we choose the probe ID showing the best e-value and higher identity (S1 Table). Expression level of each gene was obtained from different tissues by normalizing probe count, and then a global gene expression profiles were visualized by using MultiExperiment Viewer (MeV) software (v4.8.1). Some *LBD* genes with high expression level in leaf were confirmed by quantitative RT-PCR (qRT-PCR) with three independent biological replications.

### Yeast Two-Hybird Assay

Yeast two-hybrid analysis was performed in *Saccharomyces cerevisiae* strain Y2H gold according to the manufacturer’s instructions (http://www.clontech.com/). The full-length of *LjLBD11* (LBD11-Fw: CCGGggatccATGAAGGGTTATGAACCACG; LBD11-Rv: CCGGgtcgacTCAAAATATATATGGGATTTGA) and *LjLBD6* (LBD6-Fw: CCGGggatccATGGCATCATCAAGCGCTTA; LBD6-Rv: CCGGgtcgacTCATAAATTACCTCCTCCTTCAC) were cloned into the prey vector pGADT7 or the bait vector pGBKT7. The yeast cells were co-transformed with the prey vector and the bait vector using Yeast Transformation II Kit (The Epigenetics Company^TM^). Quadruple dropout medium (without adenine, histidine, leucine and trptophan) containing 200 ng/ml Aureobasidin and 40 mg/ml x-a-gal was used to test the expression of reported genes AUR1-C and MEL1.

## Results

### Identification and physical locations of *LBD* genes

A total of 45 candidate *LBD* genes were identified in *L*. *japonicus* genome based on an extensive search. Unfortunately, seven LBD proteins were excluded due to the lack of typical LOB domain or C motif (CX_2_CX_6_CX_3_C) in the N-terminus. Thus, we finally identified 38 LjLBD candidate proteins in the *L*. *Japonicus*, ranging from 95 aa to 349 aa in length. The molecular weight of LjLBD proteins varied from 10.36 kDa to 39.96 kDa, and protein pI ranged from 4.40 to 10.12 (see Table 1). For the better description in subsequent analyses, the 38 non-redundant LBD genes in *L*. *japonicus* were designated *LjLBD1*–*38* according to their positions from the top to the bottom on the chromosomes or scaffolds (Table 1). In addition, we also identify 57 *LBD* proteins in *M*. *truncatula* (named *MtLBD1-57*) based on the same criteria and found similar protein lengths and molecular weights (S1 Table). The number of *LBD* genes in these two model legumes was comparable to their homologs in other plants such as *Arabidopsis* (43 members) [1], rice (35 members) [16], maize (43 members) [17] and apple (58 members) [18].

**Table 1:** Information of *LBD* gene family identified in *Lotus japonicus*.

Gene identifier	Gene name	Genomic position	Size(aa)	MW(Da)	pI
chr1.CM0001.80.r2.a	LjLBD1	chr1:44897181–44897675	164	18162.3	6.25
chr1.CM0017.40.r2.m	LjLBD2	chr1:39247040–39248087	316	34117.4	8.65
chr1.CM0088.550.r2.d	LjLBD3	chr1:201658–202206	156	16733.2	8.3
chr1.CM0104.3290.r2.d	LjLBD4	chr1:50334865–50335833	234	25236.3	7.86
chr1.CM0125.620.r2.d	LjLBD5	chr1:16763699–16764628	190	20829.9	9.03
chr1.CM0171.410.r2.m	LjLBD6	chr1:3870813–3871385	190	20834.6	7.83
chr1.CM0375.610.r2.a	LjLBD7	chr1:56389849–56391175	205	22398.4	8.11
chr1.CM0375.630.r2.a	LjLBD8	chr1:56398498–56399492	222	24576.6	6.49
chr1.CM1255.330.r2.m	LjLBD9	chr1:31734423–31736630	184	20117.9	8.17
chr1.CM1255.380.r2.m	LjLBD10	chr1:31764240–31767714	247	25610	8.41
chr2.CM0002.640.r2.m	LjLBD11	chr2:37239732–37240286	184	20456.2	7.06
chr2.CM0008.840.r2.d	LjLBD12	chr2:23416613–23417729	220	24392.4	5.59
chr2.CM0081.1790.r2.a	LjLBD13	chr2:16470717–16471499	260	29606.1	7.97
chr2.CM0263.240.r2.m	LjLBD14	chr2:15129929–15130414	161	17933.5	7.83
chr2.LjB15M17.20.r2.m	LjLBD15	chr2:40232463–40234079	349	39956.8	7.06
chr2.LjT16G06.10.r2.d	LjLBD16	chr2:28013987–28014913	308	33953.9	7.22
chr3.CM0049.340.r2.d	LjLBD17	chr3:35912062–35912889	275	31115.4	6.63
chr3.CM0135.10.r2.m	LjLBD18	chr3:45395048–45395762	136	15523.8	8.15
chr3.CM0160.1010.r2.m	LjLBD19	chr3:37974603–37975202	199	23215.5	7.83
chr3.CM0176.110.r2.m	LjLBD20	chr3:3033466–3038666	182	20201.9	8.38
chr3.CM0246.630.r2.m	LjLBD21	chr3:30927790–30929651	203	22258.2	5.21
chr3.CM0649.90.r2.d	LjLBD22	chr3:41206303–41206659	119	13314.6	8.69
chr3.CM1488.110.r2.d	LjLBD23	chr3:1617981–1618478	165	18510.2	8.23
chr4.CM0128.420.r2.m	LjLBD24	chr4:12578313–12579139	231	25041.4	8.42
chr4.CM0229.130.r2.m	LjLBD25	chr4:11249399–11252380	174	19080.7	7.84
chr4.CM0432.3340.r2.d	LjLBD26	chr4:8660066–8660599	177	19674	5.35
chr5.CM0200.2670.r2.d	LjLBD27	chr5:33419174–33420445	199	21285	8
chr5.CM0494.160.r2.d	LjLBD28	chr5:19260103–19260417	104	11165.8	8.5
chr5.CM1667.70.r2.a	LjLBD29	chr5:29053657–29055208	223	24304.9	7.34
chr5.LjT39A22.130.r2.a	LjLBD30	chr5:31524745–31525388	130	14961	8.03
chr6.CM0738.260.r2.d	LjLBD31	chr6:5288434–5290516	167	18616.1	5.14
chr6.LjB02K20.100.r2.m	LjLBD32	chr6:5570935–5571474	179	20164	6.77
LjSGA_002536	LjLBD33	Scaffold:2417–6865	231	24409.8	7.73
LjSGA_010274	LjLBD34	Scaffold:3488–3787	99	11252.3	10.12
LjSGA_023437	LjLBD35	Scaffold:60–1891	251	27916.9	8.28
LjSGA_054888	LjLBD36	Scaffold:853–1140	95	10354.9	8.49
LjSGA_076325	LjLBD37	Scaffold:357–1193	278	30656	4.4
LjSGA_080276	LjLBD38	Scaffold:564–1165	199	22252.7	8.71

Among the 38 *LjLBD* genes, most members (84.2%) were successfully located to the six *L*. *japonicus* chromosomes, while the remaining members, *LjLBD33-38*, were localized to the six scaffolds that were not assembled into chromosomes (S1 Fig). It is obvious that the number of *LjLBD* genes on each chromosome was uneven. For instance, ten *LjLBD* members were detected on chromosome 1 followed by seven members in chromosome 3 and six in chromosome 2, whereas the fewest were found on chromosome 6 (only 2 members). Within chromosomes 4 and 5, there were only three and four *LjLBD* genes, respectively. In *Medicago truncatula*, the *LBD* genes were distributed unevenly among 8 chromosomes, and chromosome 2 contained the fewest LBD members (only 3 genes, S1 Fig).

### Sequence alignment and phylogenetic analyses

**Fig 1:**
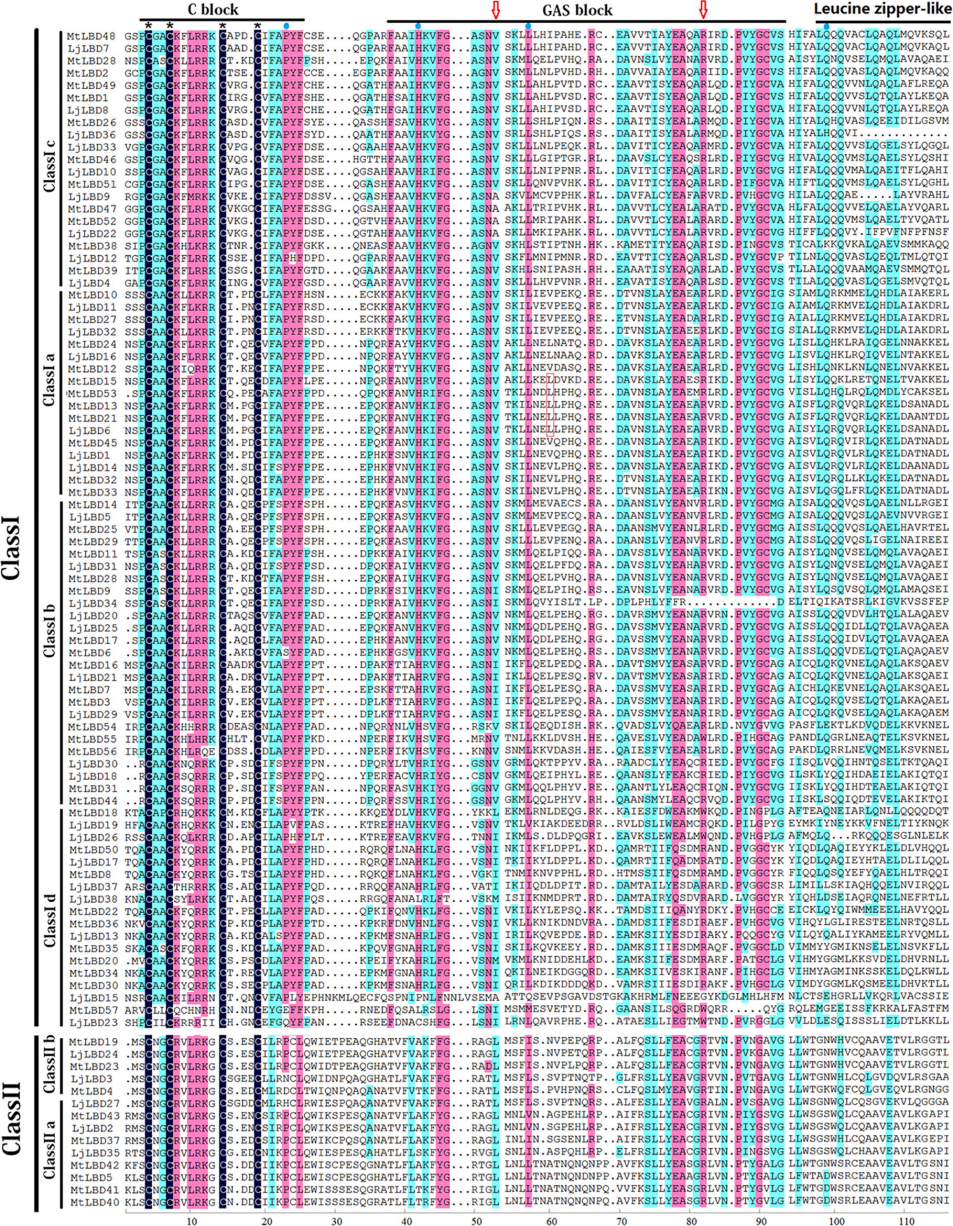
Amino acid sequence alignments of LOB domain region from *Lotus japonicus* and *Medicago truncatula*. The N-terminal LOB domain includes cysteine C block, GAS block and leucine-zipper-like regions is displayed. The valine (V) and leucine (L) residues required for motor organ specification in pea were denoted by red arrow and red frame. An arginine (R) in the GAS block required for motor organ specification was denoted by red arrow.

To identify conserved amino acid residues of LBD proteins and classify LBD members into the two classes as previously defined, we performed a sequence alignment using all of the LjLBD and MtLBD proteins. As a result, we found that all LBD proteins including 38 LjLBD and 57 MtLBD had a completely conserved CX_2_CX_6_CX_3_C motif (a C block, see Fig 1). It should be noted that the GAS block between the C block and the leucine zipper-like motif (LX_6_LX_3_LX_6_L) was also highly conserved in the LOB domain region (Fig 1). Based on the presence or absence of the leucine zipper-like motif, we further identified 33 and 48 class I LBD members, 5 and 9 class II members in *L*. *japonicus* and *M*. *truncatula*, respectively. In addition, recent study evidenced that a valine (V) residue and a leucine (L) in the GAS block, and a glutamine (Q) residue in the leucine-zipper-like motif is required for motor organ specification in pea [5]. These amino acid resides were also highly conserved in *L*. *japonicus* and *M*. *truncatula* (Fig 1). Also, an arginine (R) in GAS block region, which is very conserved in *L*. *japonicus* and *M*. *truncatula* (Fig 1), mutated into cysteine (C) in *L*. *japonicus* can lead to the defect in motor organ [5].

**Fig 2:**
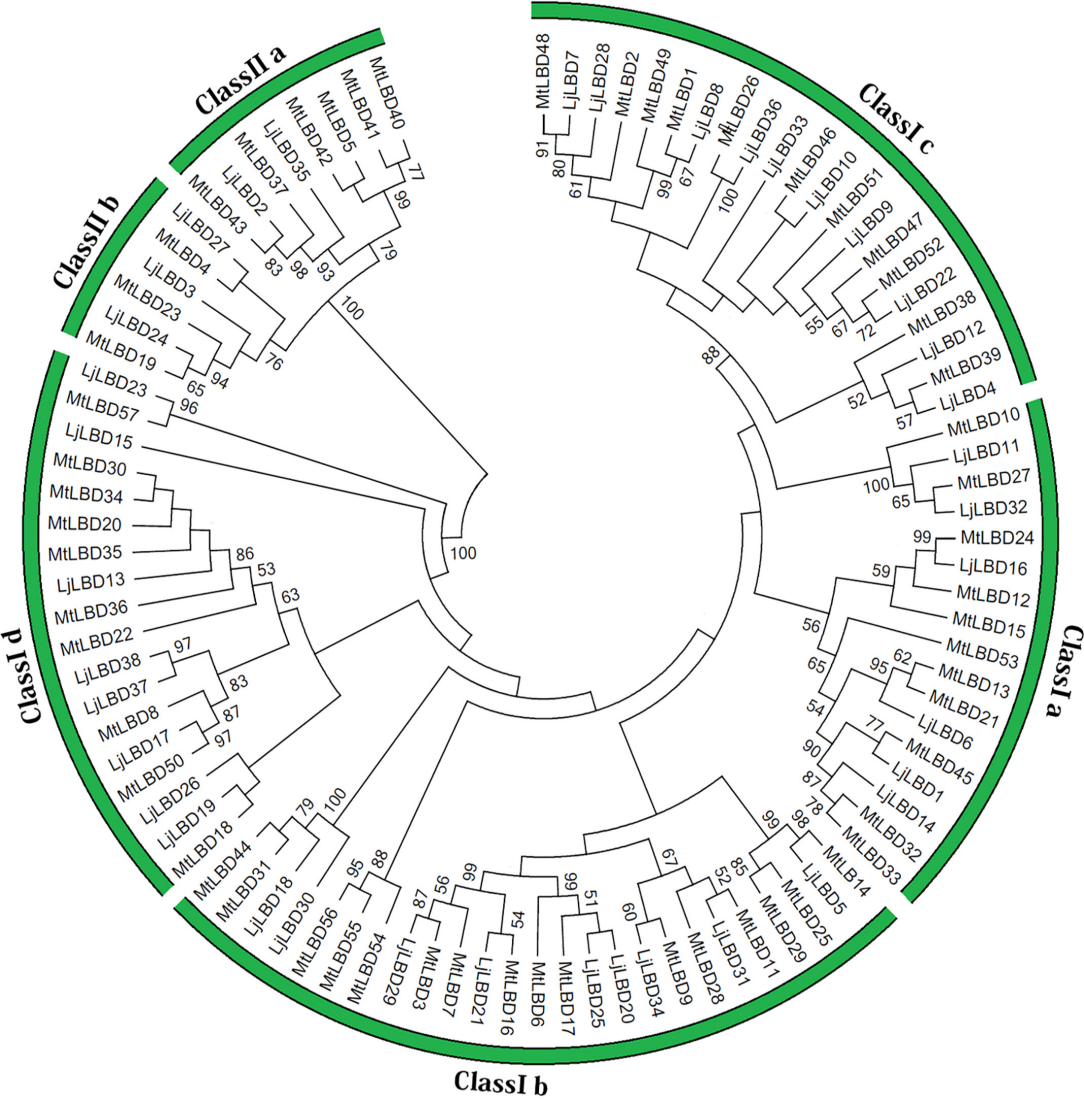
The phylogenetic tree of LBD proteins form *Lotus japonicus* and *Medicago truncatula*. The amino acid sequences of the LBD proteins were aligned with Clustal X, and the phylogenetic tree was constructed using the neighbor-joining method of MEGA 5.0 software.

According to the protein sequence alignments, we investigated the evolutionary relationships among LBD members from *L*. *japonicus* and *M*. *truncatula*. An un-rooted phylogenetic tree was constructed using the neighbor-joining method with 10,000 bootstrap replicates. Two distinct clusters were formed corresponding to the two classes in the *LBD* gene family with well supported bootstrap values as shown in Fig 2, consistent with the classification described above. Among class I, members could be further grouped into four sub-classes (Ia-Id), and class II members were divided into two sub-classes (IIa and IIb). Although three members (LjLBD15, 23 and MtLBD57) were not clustered into any subclass, we classified them into class Id subclass due to the closest genetic distances. As shown in Fig 2, two known motor organ determined genes *(MtELP1*, also called *MtLBD13* and *LjSLP*, also called *LjLBD6*) belonging to the class Ia. In addition, we found three pairs of genes duplication in *M*. *truncatula* genome including *MtLBD7* and *MtLBD16*, *MtLBD10* and *MtLBD27*, *MtLBD19* and *MtLBD23* [22]. The tree topology of LjLBD and MtLBD proteins was quite similar to the tree reported in *Arabidopsis* and rice [23]. Moreover, counterparts between *L*. *japonicus* and *M*. *truncatula* were clustered into all major clades and subclades, suggesting the functional conservation and similarity of LBD proteins in these clusters.

To detect the evolutionary relationships in the LBD family between *L*. *japonicus* and *Arabidopsis*, another un-rooted phylogenetic tree was constructed (S2 Fig). Similarly, LBD proteins from *L*. *japonicus* and *Arabidopsis* were clustered into two main clades (corresponding to class I and II), and each clade or subclade comprised members of both species, suggesting that LjLBD transcription factors were homologous with those in *Arabidopsis*. According to previously functional researches in *Arabidopsis* [23], five members from class I including *AtLBD3 (ASL9)*, *AtLBD12 (ASL5)*, *AtLBD6 (ASL2)*, *AtLBD36 (ASL1)*, *AtLOB1 (ASL4)* are involved in lateral organ (leaf and flower) development. Three genes *AtLBD16* (*ASL18*), *AtLBD18* (*ASL20*) *and AtLBD29* (*ASL16*) participate in the auxin signal transduction cascade that leads to the formation of lateral roots in *Arabidopsis*. As for the characterized class II LBD genes, three genes *AtLBD37 (ASL39)*, *AtLBD38 (ASL40)*, *AtLBD39 (ASL41)* are involved in metabolism, acting as repressors of anthocyanin synthesis and N availability signals in the plant. *AtLBD37 (ASL40)*, another class II *LBD* gene, is reported to be downregulated by gibberellin and upregulated by DELLA proteins [23]. The phylogenetic analysis indicated that the homologs in *L*. *japonicus* may have a similar function as described above.

### Functional divergence for classes and subclasses

**Fig 3:**
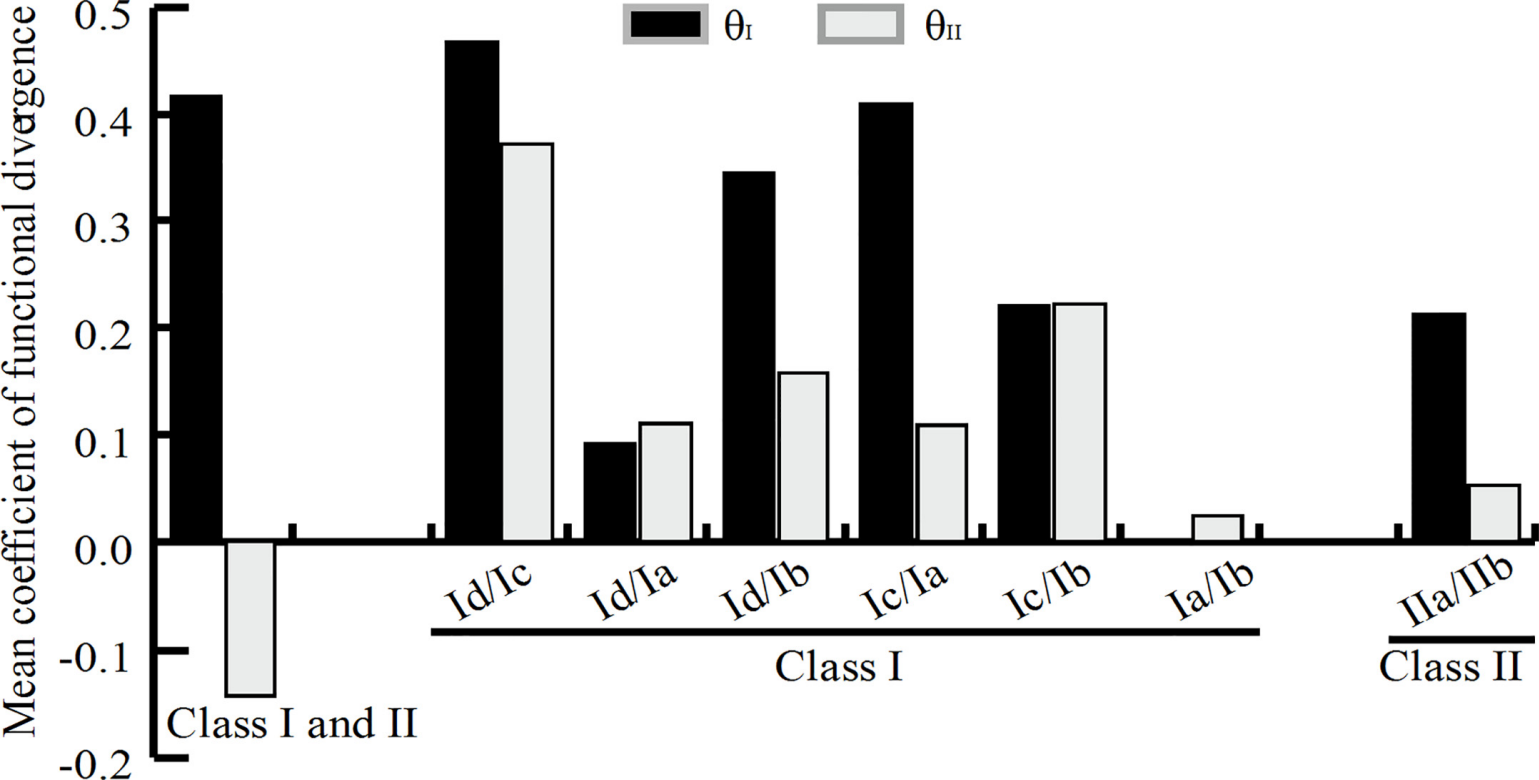
The functional divergence analysis between classes or subclasses. The estimated mean coefficients of Type-I (θI) and type-II (θII) functional divergence based on the aligned LOB domain sequences from *Lotus japonicus* and *Medicago truncatula*.

To examine whether the functional divergence between classes or subclasses was underwent in the conserved LOB domain region, we calculated the coefficients of Type-I (θ_I_) and Type-II (θ_II_) functional divergence as described in materials and methods. As shown in Fig 3, we found that the coefficient of Type-I functional divergence (θ_I_ = 0.42) between class I and class II was significant greater than 0, but the Type-II coefficient θ_II_ was negative, suggesting that significant type I functional divergence was underwent between these two classes. Moreover, we calculated the posterior probability (Q_k_) value to detect the potential amino acid residues that occurred significant changes between class I and II members. We considered 0.9 as the Q_k_ cutoff in this study and found four sites including 23P, 42H, 57L and 99Q were markedly changed, in which these four amino acid residues were highly conserved within class I but highly variable within class II (see Fig 1). Within class I, the coefficients of both θ_I_ and θ_II_ were significant between pairs Id/Ic, Id/Ia, Id/Ib, Ic/Ia and Ic/Ib, suggesting that these subclasses, particularly Id/Ic, might have experienced both Type-I and Type-II functional divergence. However, the functional divergence between Ia and Ib may be caused by the Type-II functional divergence (Fig 3). Similarly, within class II, Type-I and Type-II functional divergence were detected in the pair IIa/IIb (Fig 3).

### Conserved motifs and gene structure analyses

**Fig 4:**
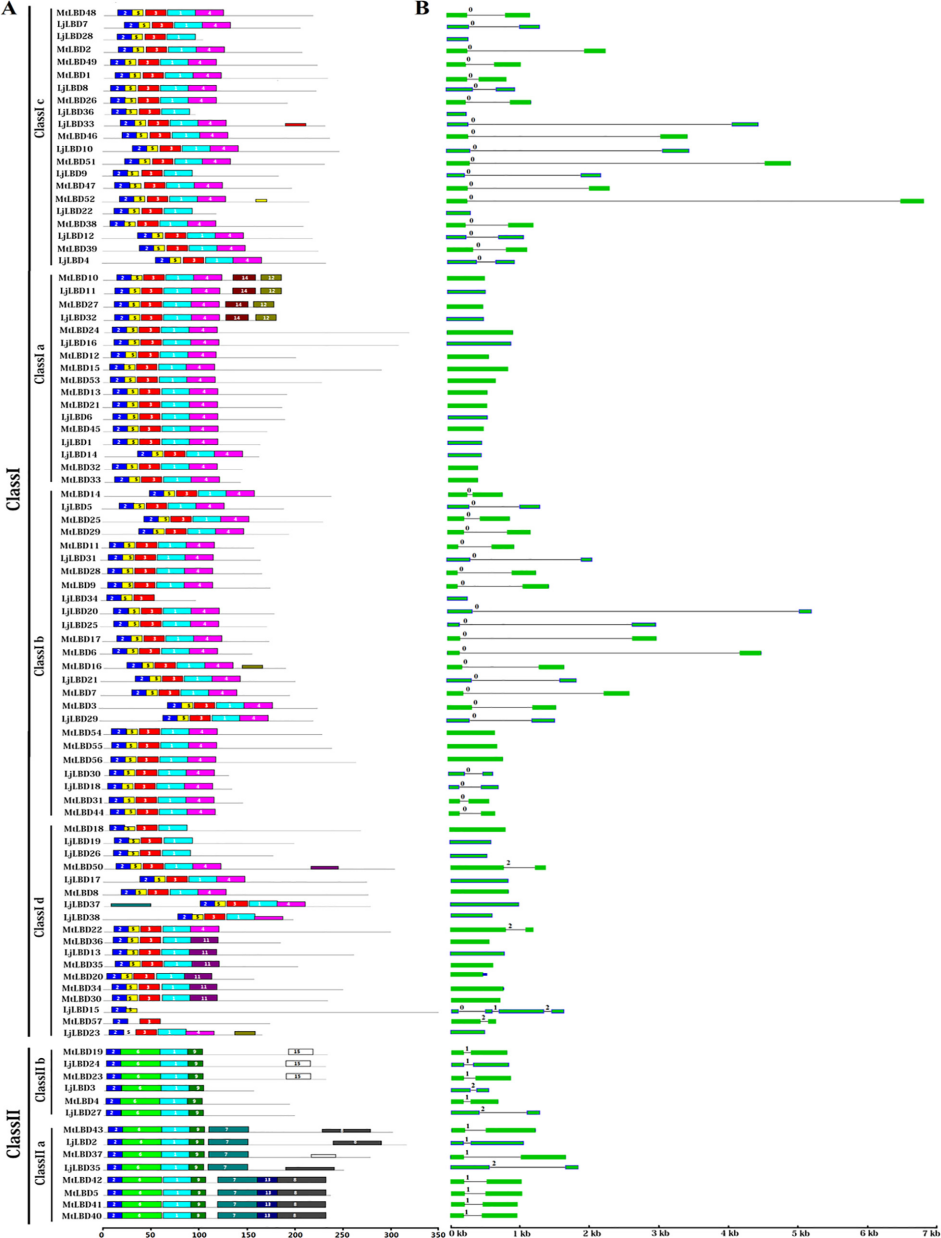
Distribution of conserved motifs and gene structure analysis of LBD gene family from *Lotus japonicus* and *Medicago truncatula*. (A) Conserved motifs analysis by MEME suite. The colorful boxes represent the different motifs 1–15. (B) The exons and introns splicing patterns. The green boxes and black lines represent the exon ans intron, respectively. The numbers indicate the intron phase. The motifs and gene sizes are indicated at the bottom of the figure.

Conserved motifs and exon/intron structures could further explore the possible evolutionary relationship of related LBD proteins. To identify the potential conserved motifs, all LBD proteins from *L*. *japonicus* and *M*. *truncatula* were subjected to the MEME suite (Fig 4A). Totally, we detected 15 high-confidence motifs (designed as motif 1–15) with significant *p* value (p < 0.001), and the consensus sequence of motifs were listed in S2 Table. Of them, motif 2 was embedded in the LOB domain that was present in all LBD proteins. Notably, the class I and class II LBD proteins harbored distinct sets of motifs. For instance, all class I proteins possessed motifs 1–5, whereas class II proteins included motifs 1, 2, 6 and 9. Further, class II proteins were split into two groups based on differential distribution of motifs 7, 13 and 8. Most of members from class I shared common motifs. In addition, some motifs were nested in specific clades. For instance, motifs 12 and 14 were shared by four members (MtLBD10, 27 and LjLBD11, 32) in class Ia; motif 11 was uniquely found in six proteins (MtLBD20, 30, 34, 35, 36 and LjLBD13) from class Id; motifs 7, 13, and 8 were specific to class IIa proteins. These findings suggest that LBD proteins with the same motifs are likely to have similar functions in plant development.

Additionally, gene structure analysis showed that almost all of the *LjLBD* genes had either one or two exons, except *LjLBD15* with four exons (see Fig 4B). We found that 20 genes included two exons, and 17 genes contained just one exon. Specifically, all *LBD* members in class Ia and most class Id members were intronless. Interestingly, while inspecting the pattern of intron insertion and splicing phase for those genes containing introns, we found that most of class I members shared splicing phase 0 (splicing occurred after the third nucleotide of the codon), whereas most of members from class II shared splicing phase 1 (splicing occurred after the first nucleotide of the codon) except three members with phase 2 (splicing occurred after the second nucleotide of the codon). Collectively, these results from conserved motifs and gene structure analyses provided additional evidence to confirm our phylogeny-based groupings.

### Expression profiles of *LBD* genes in *L*. *japonicus*

**Fig 5:**
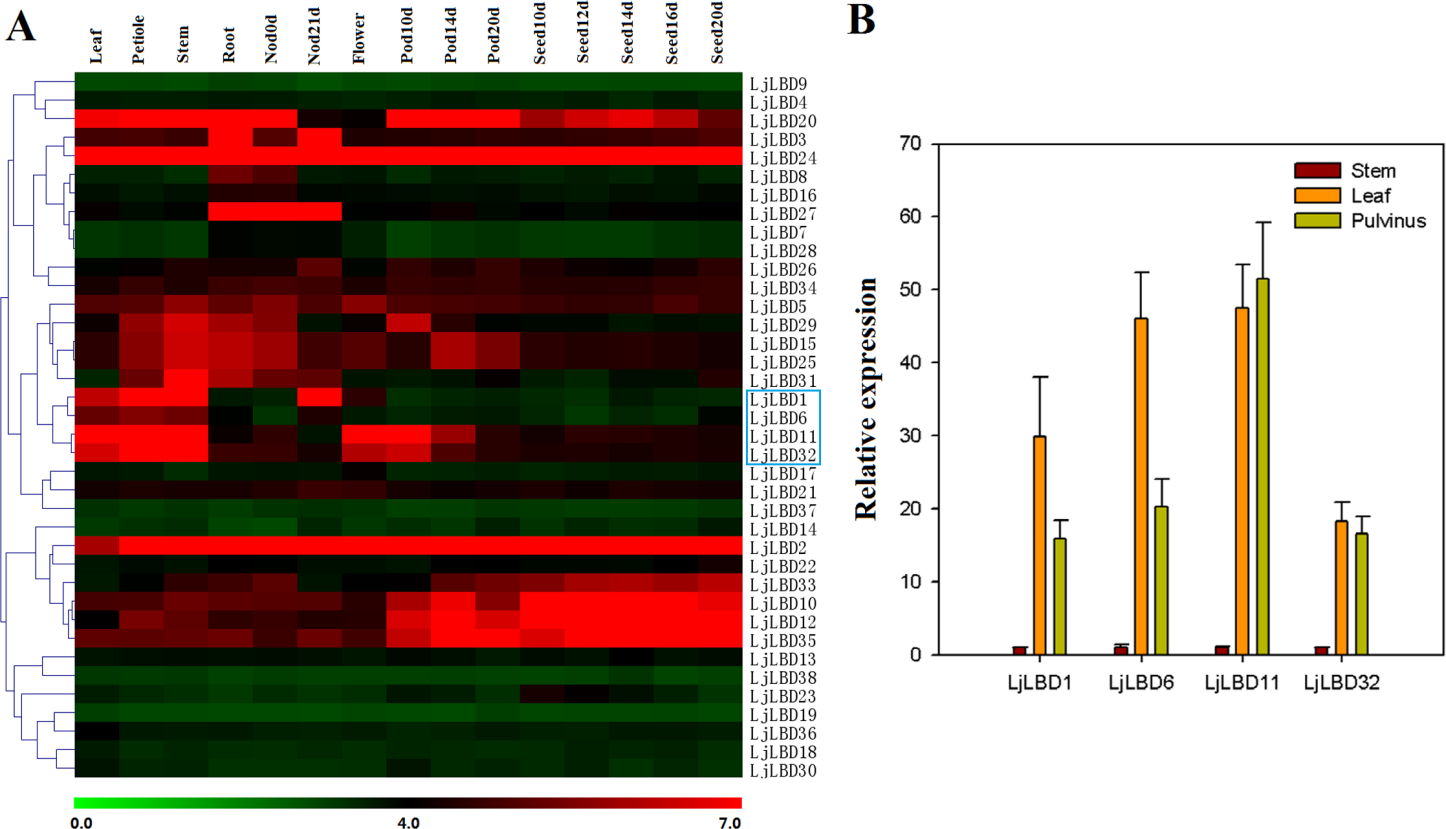
Expression profiles of LBD genes in *Lotus japonicus*. (A) Heatmap showing LBD gene expression patterns in different tissues/organs/development stages. The scale at the bottom represents log2 value. (B) Some genes highly expressed in leaf (the blue box) were confirmed by quantitative RT-PCR. The expression level of stem sample was normalized to 1.

To gain insights in understanding the potential function of *LBD* genes in *L*. *japonicus*, we investigated their expression patterns in various tissues/organs/developmental stages (Fig 5A). Transcripts for all 38 *LjLBD* genes could be detected and about half of the genes displayed high expression in at least one tissue tested. With the exception of *LjLBD2* and *LBD24* that were highly transcribed in all tissues, the remaining *LjLBD* genes exhibited tissue/organ-specific expression. Two class II genes (*LjLBD3* and *LjLBD27*) were specifically expressed in root and nodules; five genes (*LjLBD10*, *12*, *20*, *33*, *35*) showed high transcript abundance throughout pod and seed development. Also, we detected ten genes (*LjLBD1*, *2*, *5*, *6*, *10*, *11*, *20*, *24*, *32* and *35*) that were highly expressed in compound leaf and 14 members (*LjLBD1*, *2*, *3*, *5*, *10*, *15*, *20*, *25*, *26*, *27*, *29*, *31*, *33*, *35*) that were highly expressed in root nodule. These genes represented candidate genes involved in compound leaf development or root nodule formation, respectively, in which these two traits in *L*. *japonicus* are distinct phenotypic features from *Arabidopsis*. In addition, we performed hierarchical clustering based on these expression data to test whether the LBD members placed in same phylogenetic clade had similar expression patterns. Unfortunately, the result showed no clade-specific expression pattern for all *LjLBD* members.

One of main interests in this study is to identify potential LBD transcription factors involved in compound leaf or motor organ development in *L*. *japonicus*. Thus, we selected a set of genes that were highly expressed in leaf but not constitutively expressed, and examined their expression levels in stem, compound leaf and pulvinus using qRT-PCR technique. Results showed that all selected four genes including LjLBD1,6,11 and 32 had high expression levels in leaf and pulvinus tissue but were quite low expression in stem (Fig 5B), suggesting the potential roles in compound leaf and pulvinus development.

### The interaction of *LjLBD11* and *LjLBD6*

**Fig 6:**
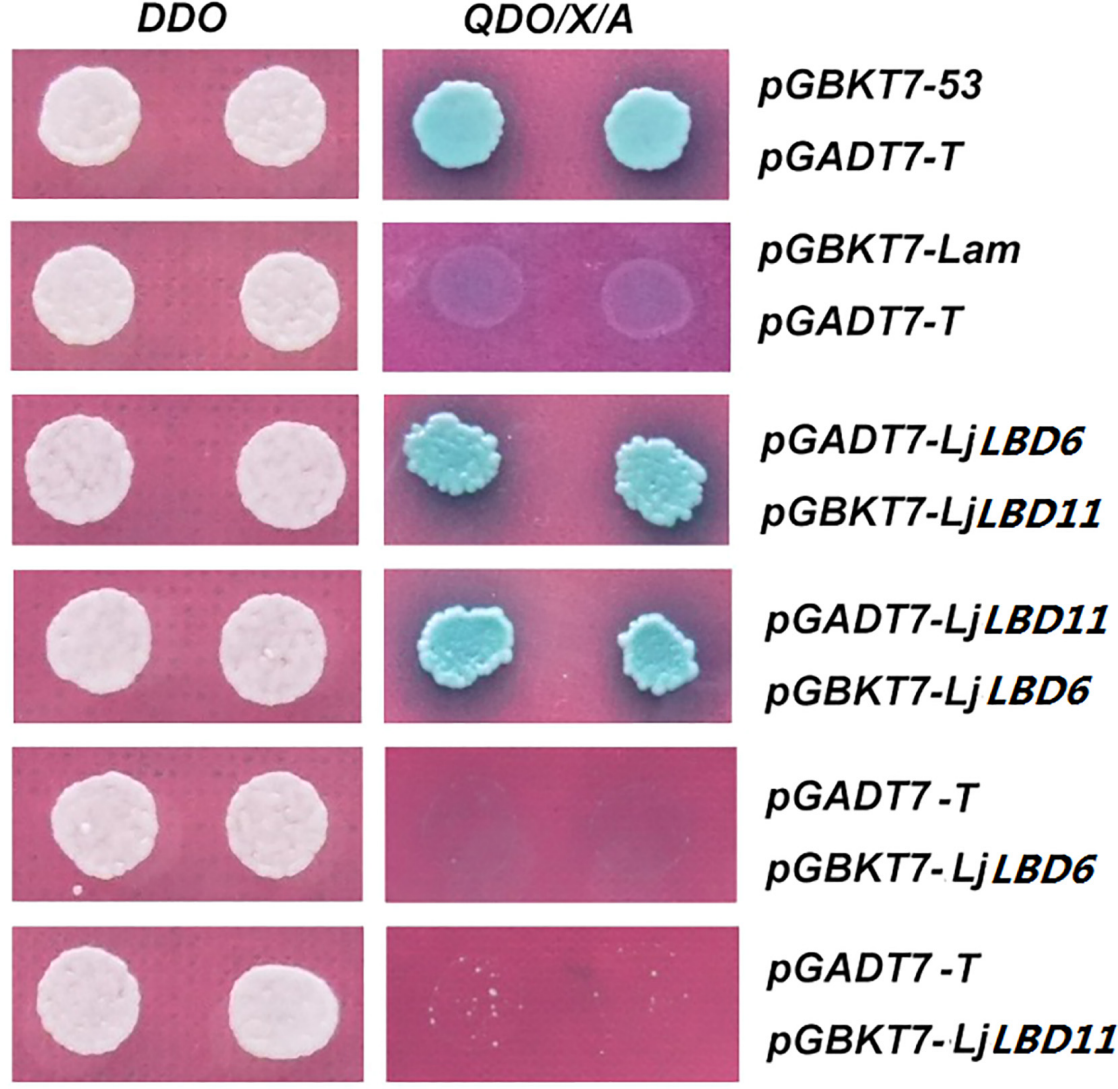
Interaction between LjLBD11 and LjLBD6 protein by yeast two-hybird assay. pGBKT7-53 in combination with pGADT7-T was used as a positive control and pGBKT7-Lam with pGADT7-T was used as negative control. Yeast grew on DDO medium to select for both the bait and prey proteins (left). QDO/X/A media allow the growth of only positively interacting clones (right).

Based on the expression analysis in this study and previous study [4], *LjLBD6* and *LjLBD11* (responding to *LjLOB3* and *LjLOB1*) had a strong expression at the bases of leaflet primordia and considered likely to have roles in compound leaf development. Moreover, mutation of *LjLBD6* results in loss of motor organs in *L*. *Japonicus* [5] that was in good agreement with it expression in pulvinus (Fig 5). It is should be noted that *LjLBD11* gene exhibited more high expression level in compound leaf and pulvinus relative to *LjLBD6*. However, the functional roles of *LjLBD11* in compound development and motor organs formation remained unknown. In addition, previous studies uncovered that LBD proteins can usually form the Homo- or heter-dimerization through the leucine-zipper-like motif located at the C-terminus of the LBD domain [3]. Therefore, we speculated that *LjLBD6* and *LjLBD11* might exert their function via a protein complex. To test this, the protein interaction between LjLBD6 and LjLBD11 was performed by yeast two-hybird experiment. The yeast strain was co-transformed with the indicated combinations of *LjLBD6* and *LjLBD11* fused to the GAL4 activition domain (AD) and GAL4 DNA-binding domain (BD), respectively. Among the clones grown on the dropout medium lacking tryptophan, leucine, histidine and adenine, we found that clones containing *LjLBD6* and *LjLBD11* showed growth (see Fig 6). This result showed that LjLBD11 protein can interact with LjLBD6 protein, suggesting that the LjLBD11 may play a similar function of LjLBD6 involved in compound leaf development or specification of motor organ identity, but the detail biological function remains to be determined.

## Discussion

The plant-specific LBD (Lateral Organ Boundaries domain) transcription factors play critical roles in the control of plant development, in particular in lateral organ development. The *LBD* gene family has been extensively studied in diverse species [1,16–18] but very little is known in legumes. Legumes are models for studying development of some specific features absent in other model plants such as *Arabidopsis*, including compound leaves, motor organs, and root nodules. Thus, studies on *LBD* genes in legumes would provide the basis for unraveling mechanisms of plant development that are currently not understood.

In this study, we identified 38 and 57 putative LBD transcription factors in *L*. *japonicus* and *M*. *truncatula* respectively, both of which are important model plants in legumes. Compared with other plants, *L*. *japonicus* and *M*. *truncatula* apparently harbored more *LBD* members in its genome, probably due to genome duplication that resulted in gene family expansion during evolution. All LBD proteins identified in this study contained a highly conserved CX_2_CX_6_CX_3_C zinc finger-like domain, implying its structural and functional necessity. Among class I members, most proteins contained an additional motif (LX_6_LX_3_LX_6_L) in the C-terminus, which has been demonstrated to function in protein-protein interaction [2]. Interestingly, analysis of conserved motifs outside the LOB domain found that members of the two classes (class I and II) harbored different motifs. The phylogenetic tree of LBD transcription factors from *L*. *japonicus*, *M*. *truncatula* and *Arabidopsis* was distinctly split into two clusters, corresponding to class I and class II members. Gene structure and motif analyses further supported phylogenetic tree analysis similar to the previous reports in *Arabidopsis*, rice, maize and apple [17,18,23], indicating that the *LBD* gene family may be highly conserved among species. In addition, functional divergence analysis showed that significant type I functional divergence was detected between classes I and II, and four amino acid residues with site-specific changes in evolutionary rates may have a main contribution.

To understand the functions of *LBD* genes in *L*. *japonicus*, we examined their expression profiles in different tissues/organ/developmental stages. For example, *AtLBD37* (AT5G67420), *AtLBD38* (AT3G49940) and *AtLBD39* (AT4G37540) function in the regulation of plant basic metabolism in *Arabidopsis* [12], and their homologous gene *LjLBD24* in *L*. *japonicus* exhibited constitutive expression, suggesting that it may also have a basic function in plant growth and development. For three genes (*LjLBD7*, *LjLBD8* and *LjLBD28*) with relatively high expression in root, their homologs in *Arabidopsis* (AtLBD16, AtLBD18 and AtLBD29) represent a set of auxin-regulated genes that also display root-specific expression [7,8,9], strongly implying that these three genes may be involved in lateral root formation in *L*. *japonicus*. The *LjLBD2* was expressed in all tissues tested in this study, while its homolog *AtLBD40* is reported to respond to gibberellin [11]. *Arabidopsis AtLBD30* is thought to regulate embryogenesis and floral development, and its homologs *LjLBD10* and *LjLBD33* were had abundant transcripts in flower and seed developmental stages [24,25], suggesting that they probably have similar functions. In addition, we noticed that both *LjLBD11* and *LjLBD6* were strongly expressed in compound leaf, in good agreement with previous results from RNA in situ hybridization. Previous study showed that *LjLBD6* may play a conserved role in genetic determinant of motor organ identity in legumes [5]. By the Yeast two-hybrid experiment, we further found that LjLBD6 can interact with LjLBD11 at the protein level, indicating that LjLBD11 protein may have similar important function in the control of compound leaf development and determination of motor organ identity. Due to the lack of a *LjLBD11* mutant in *L*. *japonicus*, the detailed function of *LjLBD11* gene remains unknown, but worth further inquiry using other methods such as siRNA-mediated silencing.

In summary, the current study defined in detail the *LBD* gene family in legumes based on genome sequences. The gene structure, conserved motif, and phylogenetic analyses indicated that the functions of *LBD* genes are likely conserved among angiosperms. In addition, our results indicate that the Type I functional divergence with some site-specific shifts may be the main force for between class I and II. Importantly, The expression patterns of *LjLBD11* and its interaction with *LjLBD6* provided the molecular basis for the mechanisms underlying *LjLBD11* gene in compound leaf development, motor organ specification in *L*. *japonicus*, even more generally in legumes.

## Supporting Information

S1 Fig The chromosomal distribution of the LBD gene family in *Lotus japonicus* and *Medicago truncatula*. The chromosome number is indicated at the bottom of each chromosome. Genes without intron are marked with red asterisk. Segmental duplication genes in *M*. *truncatula* are linked by red dash lines.
(TIF)

S2 Fig The phylogenetic analysis of LBD members from *Lotus japonicus* and *Arabidopsis*. The amino acid sequences of the LBD proteins were aligned with Clustal X, and the phylogenetic tree was constructed using the neighbor-joining method of MEGA 5.0 software. The red clade represents the Class II members. The LBD proteins in bracket meant that they have been investigated in other studies.
(TIF)

S1 Table Information of LBD gene family identified in *Medicago truncatula*. (DOCX)

S2 Table Motif sequences identified by MEME tools. (DOCX)

## References

[1] Shuai B, Reynaga-Peña CG, Springer PS. The lateral organ boundaries gene defines a novel, plant-specific gene family. Plant Physiol. 2002; 129(2): 747–761. 12068116 10.1104/pp.010926 PMC161698

[2] Matsumura Y, Iwakawa H, Machida Y, Machida C. Characterization of genes in the *ASYMMETRIC LEAVES2/LATERAL ORGAN BOUNDARIES* (*AS2/LOB*) family in *Arabidopsis thaliana*, and functional and molecular comparisons between *AS2* and other family members. Plant J. 2009; 58(3): 525–537. 10.1111/j.1365-313X.2009.03797.x 19154202 PMC2721968

[3] Husbands A, Bell EM, Shuai B, Smith HM, Springer PS. LATERAL ORGAN BOUNDARIES defines a new family of DNA-binding transcription factors and can interact with specific bHLH proteins. Nucleic Acids Res. 2007; 35(19): 6663–6671. 17913740 10.1093/nar/gkm775 PMC2095788

[4] Luo JH, Weng L, Luo D. Isolation and expression pattern of *LATERAL ORGAN B*OUNDARIES-like genes in *Lotus japonicas*. J Plant Physiol Mol Biol. 2006; 32(2): 202–208. 16622320

[5] Chen J, Moreau C, Kawaquchi M, Hofer J, Ellis N, Chen R. Conserved genetic determinant of motor organ identity in *Medicago truncatula* and related legumes. Proc Natl Acad Sci USA. 2012; 109(29): 11723–11728. 10.1073/pnas.1204566109 22689967 PMC3406810

[6] Semiarti E, Ueno Y, Tsukaya H, Iwakawa H, Machida C, Machida Y. The *ASYMMETRIC LEAVES2* gene of *Arabidopsis thaliana* regulates formation of a symmetric lamina, establishment of venation and repression of meristem-related homeobox genes in leaves. Development. 2001; 128(10): 1771–1783. 11311158 10.1242/dev.128.10.1771

[7] Lee HW, Kim NY, Lee DJ, Kim J. LBD18/ASL20 regulates lateral root formation in combination with LBD16/ASL18 downstream of ARF7 and ARF19 in *Arabidopsis*. Plant Physiol. 2009; 151(3): 1377–1389. 10.1104/pp.109.143685 19717544 PMC2773067

[8] Okushima Y, Overvoorde PJ, Arima K, Alonso JM, Chan A, Chang C et al Functional genomic analysis of the *AUXIN RESPONSE FACTOR* gene family members in *Arabidopsis thaliana*: unique and overlapping functions of *ARF7* and ARF19. Plant Cell. 2005; 17(2): 444–463. 15659631 10.1105/tpc.104.028316 PMC548818

[9] Okushima Y, Fukaki H, Onoda M, Theologis A, Tasaka M. ARF7 and ARF19 regulate lateral root formation via direct activation of *LBD/ASL* genes in *Arabidopsis*. Plant Cell. 2007; 19(1): 118–130. 17259263 10.1105/tpc.106.047761 PMC1820965

[10] Naito T, Yamashino T, Kiba T, Koizumi N, Kojima M, Sakakibara H et al A link between cytokinin and ASL9 (ASYMMETRIC LEAVES 2 LIKE 9) that belongs to the AS2/LOB (LATERAL ORGAN BOUNDARIES) family genes in *Arabidopsis thaliana*. Biosci Biotechnol Biochem. 2007; 71(5): 1269–1278. 17485849 10.1271/bbb.60681

[11] Zentella R, Zhang ZL, Park M, Thomas SG, Endo A, Murase K, et al Global analysis of DELLA direct targets in early gibberellins signaling in Arabidopsis. Plant Cell. 2007; 19(10): 3037–3057. 17933900 10.1105/tpc.107.054999 PMC2174696

[12] Rubin G, Tohge T, Matsuda F, Saito K, Scheible WR. Members of the LBD family of transcription factors repress anthocyanin synthesis and affect additional nitrogen responses in *Arabidopsis*. Plant Cell. 2009; 21(11): 3567–3584. 10.1105/tpc.109.067041 19933203 PMC2798321

[13] Bortiri E, Chuck G, Vollbrecht E, Rocheford T, Martienssen R, Hake S. *ramosa2* encodes a LATERAL ORGAN BOUNDARY domain protein that determines the fate of stem cells in branch meristems of maize. Plant Cell. 2006; 18(3): 574–585. 16399802 10.1105/tpc.105.039032 PMC1383634

[14] Xu C, Tai H, Saleem M, Ludwig Y, Majer C, Berendzen KW et al Cooperative action of the paralogous maize lateral organ boundaries (LOB) domain proteins RTCS and RTCL in shoot-borne root formation. New Phytol. 2015; 207(4): 1123–1133. 10.1111/nph.13420 25902765

[15] Liu H, Wang S, Yu X, Yu J, He X, Zhang S, et al ARL1, a LOB-domain protein required for adventitious root formation in rice. Plant J. 2005; 43(1); 47–56. 15960615 10.1111/j.1365-313X.2005.02434.x

[16] Yang Y, Yu X, Wu P. Comparison and evolution analysis of two rice subspecies LATERAL ORGAN BOUNDARIES domain gene family and their evolutionary characterization from Arabidopsis. Mol Phylogenet Evol. 2006; 39(1): 248–262. 16290186 10.1016/j.ympev.2005.09.016

[17] Zhang YM, Zhang SZ, Zheng CC. Genomewide analysis of LATERAL ORGAN BOUNDARIES Domain gene family in Zea mays. J Genet. 2014; 93(1): 79–91. 24840825 10.1007/s12041-014-0342-7

[18] Wang X, Zhang S, Su L, Liu X, Hao Y. A genome-wide analysis of the LBD (LATERAL ORGAN BOUNDARIES domain) gene family in Malus domestica with a functional characterization of MdLBD11. PLoS One. 2013; 8(2): e57044 10.1371/journal.pone.0057044 23468909 PMC3585328

[19] Tamura K, Peterson D, Peterson N, Stecher G, Nei M, Kumar S. MEGA5: molecular evolutionary genetics analysis using maximum likelihood, evolutionary distance, and maximum parsimony methods. Mol Biol Evol. 2011; 28(10): 2731–2739. 10.1093/molbev/msr121 21546353 PMC3203626

[20] Gu X, Zou Y, Su Z, Huang W, Zhou Z, Arendsee Z, et al An update of DIVERGE software for functional divergence analysis of protein family. Mol Biol Evol. 2013; 30(7): 1713–1719. 10.1093/molbev/mst069 23589455

[21] Verdier J, Torres-Jerez I, Wang M, Andriankaja A, Allen SN, He J, et al Establishment of the Lotus japonicus Gene Expression Atlas (LjGEA) and its use to explore legume seed maturation. Plant J. 2013; 74(2): 351–362. 10.1111/tpj.12119 23452239

[22] Young ND, Debellé F, Oldroyd GE, Geurts R, Cannon SB, Udvardi MK, et al The Medicago genome provides insight into the evolution of rhizobial symbioses. Nature. 2011; 480(7378): 520–524. 10.1038/nature10625 22089132 PMC3272368

[23] Majer C, Hochholdinger F. Defining the boundaries: structure and function of LOB domain proteins. Trends Plant Sci. 2011; 16(1): 47–52. 10.1016/j.tplants.2010.09.009 20961800

[24] Borghi L, Bureau M, Simon R. Arabidopsis JAGGED LATERAL ORGANS is expressed in boundaries and coordinates KNOX and PIN activity. Plant Cell. 2007; 19(6): 1795–1808. 17557810 10.1105/tpc.106.047159 PMC1955719

[25] Soyano T, Thitamadee S, Machida Y, Chua NH. ASYMMETRIC LEAVES2–LIKE19/LATERAL ORGAN BOUNDARIES DOMAIN30 and ASL20/LBD18 regulate tracheary element differentiation in Arabidopsis. Plant Cell. 2008; 20(12): 3359–3373. 10.1105/tpc.108.061796 19088331 PMC2630433

