# Supplementary figures and images for: Genome-Wide Identification, Evolutionary Analysis and Expression Profiles of LATERAL ORGAN BOUNDARIES DOMAIN Gene Family in Lotus japonicus and Medicago truncatula
Source: PLoS One. 2016 Aug 25;11(8):e0161901. doi: 10.1371/journal.pone.0161901 (PMC4999203; doi:10.1371/journal.pone.0161901)

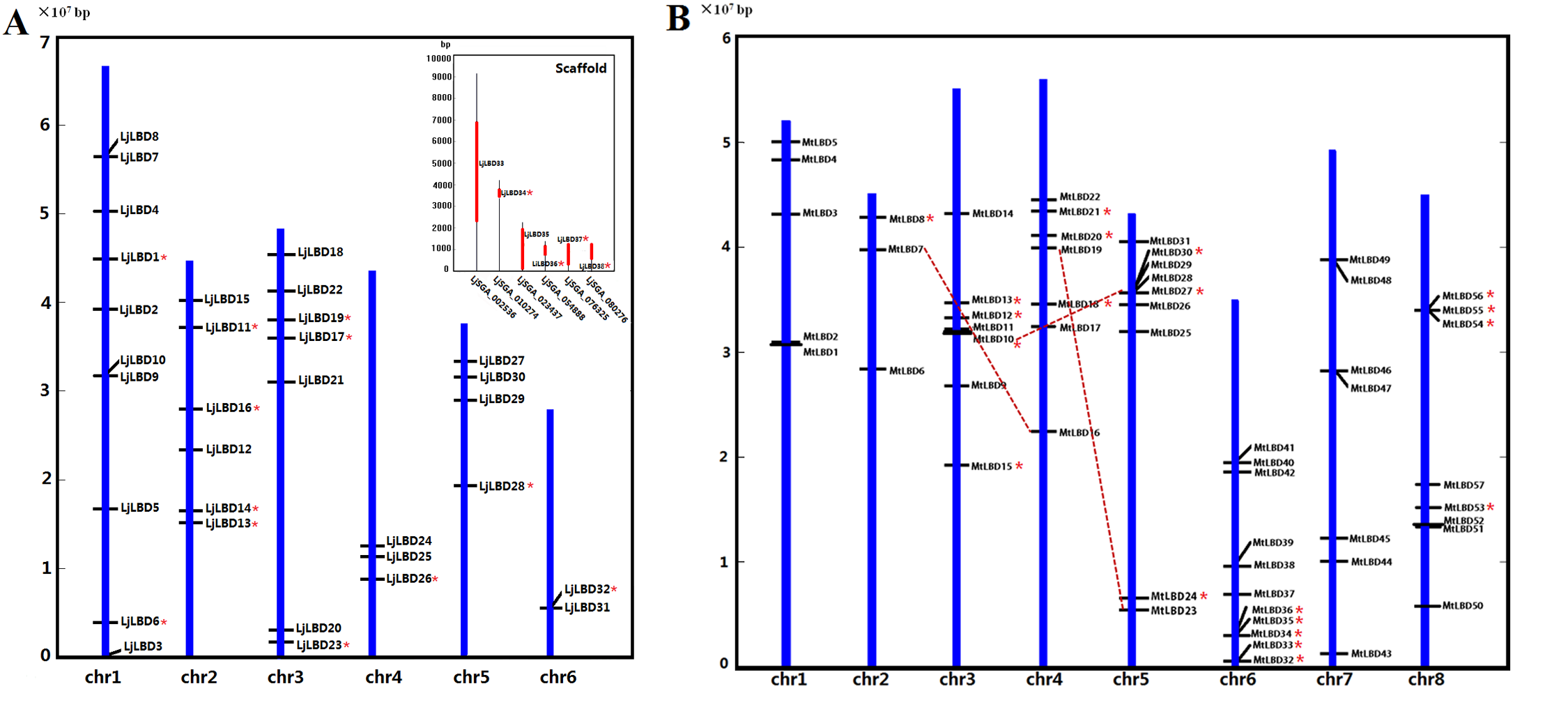

Supplement: S1 Fig — The chromosome number is indicated at the bottom of each chromosome. Genes without intron are marked with red asterisk. Segmental duplication genes in M. truncatula are linked by red dash lines. (TIF) [file pone.0161901.s001.tif]

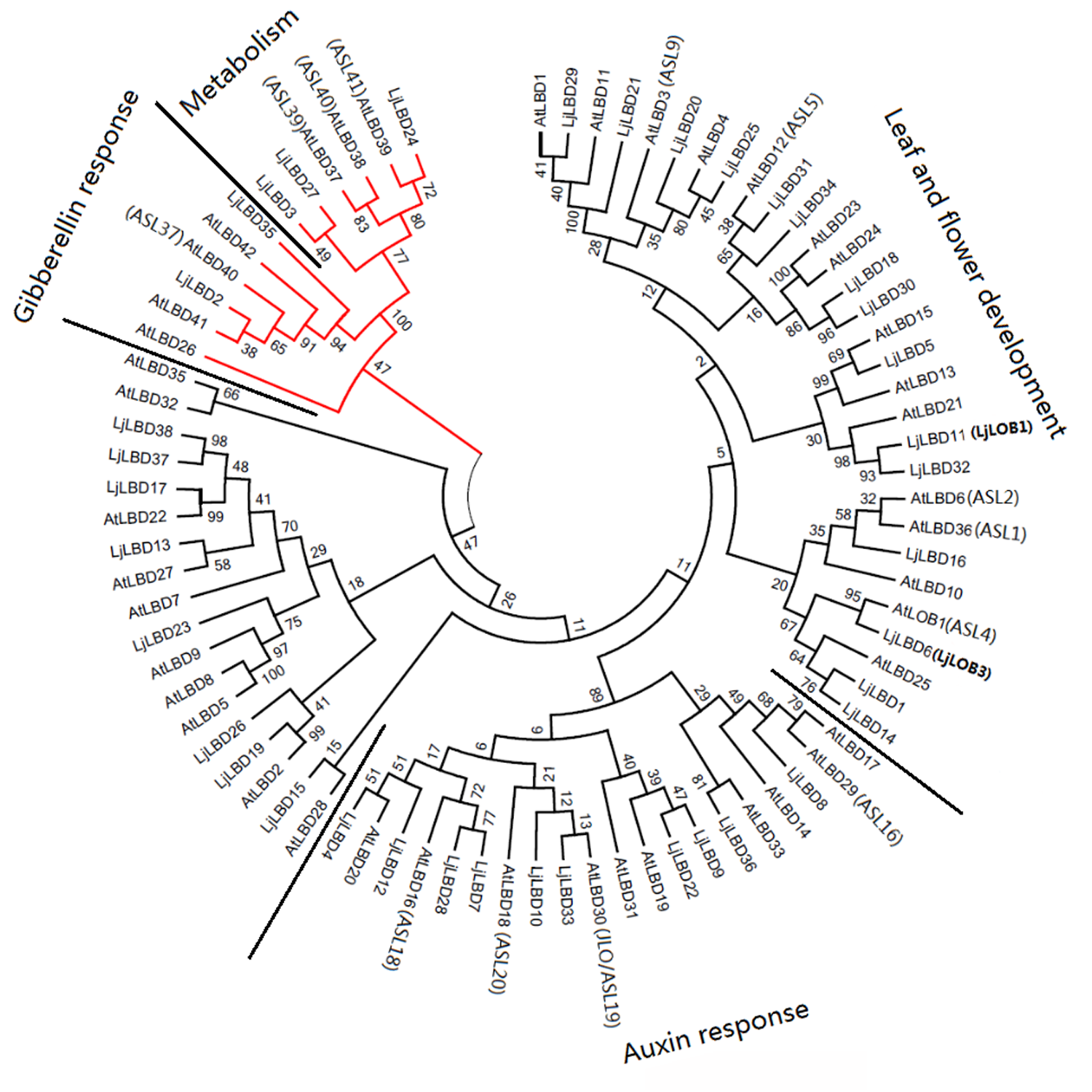

Supplement: S2 Fig — The amino acid sequences of the LBD proteins were aligned with Clustal X, and the phylogenetic tree was constructed using the neighbor-joining method of MEGA 5.0 software. The red clade represents the Class II members. The LBD proteins in bracket meant that they have been investigated in other studies. (TIF) [file pone.0161901.s002.tif]
