# Supplementary material for: Genome-Wide Identification, Evolutionary Analysis and Expression Profiles of LATERAL ORGAN BOUNDARIES DOMAIN Gene Family in Lotus japonicus and Medicago truncatula
Source: PLoS One. 2016 Aug 25;11(8):e0161901. doi: 10.1371/journal.pone.0161901 (PMC4999203; doi:10.1371/journal.pone.0161901)
Supplement: S1 Table — (DOCX) [file pone.0161901.s003.docx]

**S1 Table.** **Information of LBD gene family identified in *Medicago truncatula*.**

| Gene identifier | Gene name | Genomic position | Size(aa) | MW(Da) | pI |
| --- | --- | --- | --- | --- | --- |
| Medtr1g070205 | MtLBD1 | chr1:30903939-30904815 | 234 | 26107.8 | 5.77 |
| Medtr1g070220 | MtLBD2 | chr1:30927170-30924861 | 207 | 22857.1 | 7.86 |
| Medtr1g095850 | MtLBD3 | chr1:43142347-43140778 | 229 | 25124.9 | 6.91 |
| Medtr1g106420 | MtLBD4 | chr1:48276270-48276962 | 194 | 21638.4 | 8 |
| Medtr1g110840 | MtLBD5 | chr1:50042373-50043391 | 237 | 26191 | 9.08 |
| Medtr2g068760 | MtLBD6 | chr2:28560680-28556166 | 159 | 17724.9 | 8.49 |
| Medtr2g093310 | MtLBD7 | chr2:39790195-39792818 | 198 | 22119 | 4.65 |
| Medtr2g100020 | MtLBD8 | chr2:42933203-42934233 | 277 | 31723.1 | 5.44 |
| Medtr3g031660 | MtLBD9 | chr3:26664728-26666203 | 177 | 19833.4 | 5.66 |
| Medtr3g071420 | MtLBD10 | chr3:32036594-32036037 | 185 | 20570.6 | 7.72 |
| Medtr3g071590 | MtLBD11 | chr3:32140217-32141188 | 159 | 17933.6 | 7.84 |
| Medtr3g073690 | MtLBD12 | chr3:33246574-33245882 | 202 | 22606.7 | 8.8 |
| Medtr3g077240 | MtLBD13 | chr3:34675184-34675762 | 192 | 20821.4 | 7.86 |
| Medtr3g094690 | MtLBD14 | chr3:43201277-43200336 | 237 | 26174.8 | 8.58 |
| Medtr3g452660 | MtLBD15 | chr3:19281985-19282860 | 291 | 31517.1 | 5.83 |
| Medtr4g060950 | MtLBD16 | chr4:22428224-22426541 | 190 | 20737.4 | 4.93 |
| Medtr4g083680 | MtLBD17 | chr4:32513279-32510274 | 174 | 18952.3 | 7.89 |
| Medtr4g088035 | MtLBD18 | chr4:34669124-34669930 | 268 | 30120.1 | 7.99 |
| Medtr4g095600 | MtLBD19 | chr4:39872592-39871782 | 233 | 25403 | 7.67 |
| Medtr4g099230 | MtLBD20 | chr4:41121940-41122410 | 156 | 17291.8 | 8.13 |
| Medtr4g105170 | MtLBD21 | chr4:43583170-43583736 | 188 | 20628.3 | 7.86 |
| Medtr4g107450 | MtLBD22 | chr4:44467992-44466801 | 299 | 33969.8 | 5.34 |
| Medtr5g015880 | MtLBD23 | chr5:5587319-5588190 | 231 | 25123.8 | 8.09 |
| Medtr5g017950 | MtLBD24 | chr5:6650084-6651040 | 318 | 34853.8 | 7.09 |
| Medtr5g075020 | MtLBD25 | chr5:31869791-31868647 | 230 | 24832.2 | 8.71 |
| Medtr5g080470 | MtLBD26 | chr5:34446659-34447881 | 192 | 21179.6 | 6 |
| Medtr5g083010 | MtLBD27 | chr5:35808952-35808419 | 177 | 19801.5 | 7.24 |
| Medtr5g083230 | MtLBD28 | chr5:35918706-35919994 | 167 | 18806.3 | 7 |
| Medtr5g083960 | MtLBD29 | chr5:36233064-36231692 | 196 | 21826.7 | 7.41 |
| Medtr5g085390 | MtLBD30 | chr5:36914978-36915682 | 234 | 26314.6 | 5.31 |
| Medtr5g093010 | MtLBD31 | chr5:40608170-40607599 | 145 | 16601.9 | 8.67 |
| Medtr6g005070 | MtLBD32 | chr6:591104-591541 | 145 | 15925.9 | 7.07 |
| Medtr6g005080 | MtLBD33 | chr6:600678-601106 | 142 | 15838.9 | 6.77 |
| Medtr6g011200 | MtLBD34 | chr6:3080882-3080130 | 250 | 28203.6 | 4.73 |
| Medtr6g011230 | MtLBD35 | chr6:3094208-3093600 | 202 | 22870.2 | 7.74 |
| Medtr6g011250 | MtLBD36 | chr6:3104219-3104773 | 184 | 20980.7 | 4.8 |
| Medtr6g018270 | MtLBD37 | chr6:6985847-6984183 | 278 | 30525.6 | 7.64 |
| Medtr6g027700 | MtLBD38 | chr6:9670716-9669467 | 209 | 23186.2 | 8.81 |
| Medtr6g027710 | MtLBD39 | chr6:9674698-9673541 | 227 | 24421.4 | 9.49 |
| Medtr6g054080 | MtLBD40 | chr6:19556243-19555283 | 231 | 25430 | 9.27 |
| Medtr6g054150 | MtLBD41 | chr6:19583540-19582583 | 231 | 25573.3 | 9.83 |
| Medtr6g453130 | MtLBD42 | chr6:18686374-18687395 | 231 | 25275.8 | 9.08 |
| Medtr7g007010 | MtLBD43 | chr7:1307968-1306755 | 301 | 32936.4 | 7.99 |
| Medtr7g028905 | MtLBD44 | chr7:10005506-10006169 | 117 | 13461.4 | 9.03 |
| Medtr7g033800 | MtLBD45 | chr7:12360924-12360406 | 172 | 18676.9 | 5.61 |
| Medtr7g074990 | MtLBD46 | chr7:28119114-28122591 | 237 | 24876.9 | 8.49 |
| Medtr7g075230 | MtLBD47 | chr7:28102339-28099993 | 197 | 21929.8 | 7.03 |
| Medtr7g096530 | MtLBD48 | chr7:38731270-38732479 | 218 | 24173.3 | 7.43 |
| Medtr7g096610 | MtLBD49 | chr7:38752769-38751686 | 224 | 25086.2 | 6.33 |
| Medtr8g017090 | MtLBD50 | chr8:5747582-5746214 | 303 | 34385.1 | 4.99 |
| Medtr8g036085 | MtLBD51 | chr8:13270811-13265860 | 232 | 24712 | 7.69 |
| Medtr8g036105 | MtLBD52 | chr8:13286739-13293624 | 216 | 23579.7 | 7.55 |
| Medtr8g040900 | MtLBD53 | chr8:15262574-15263260 | 228 | 25002.9 | 8.4 |
| Medtr8g079580 | MtLBD54 | chr8:34134551-34133865 | 228 | 25189.1 | 5.88 |
| Medtr8g079620 | MtLBD55 | chr8:34145469-34144753 | 238 | 25825.4 | 6.59 |
| Medtr8g079660 | MtLBD56 | chr8:34155809-34155015 | 264 | 28266.8 | 5.31 |
| Medtr8g445680 | MtLBD57 | chr8:17428720-17428083 | 174 | 19912 | 5.34 |
