# Supplementary material for: Genome-Wide Identification, Evolutionary Analysis and Expression Profiles of LATERAL ORGAN BOUNDARIES DOMAIN Gene Family in Lotus japonicus and Medicago truncatula
Source: PLoS One. 2016 Aug 25;11(8):e0161901. doi: 10.1371/journal.pone.0161901 (PMC4999203; doi:10.1371/journal.pone.0161901)
Supplement: S2 Table — (DOCX) [file pone.0161901.s004.docx]

**S2 Table. Motif sequences identified by MEME tools**

| **Motif** | **Width** | **Multilevel consensus sequence** |
| --- | --- | --- |
| 1 | 29 | QREDAVNSLVYEAEARIRDPVYGCVGHIW |
| 2 | 15 | CAACKFLRRKCTPDC |
| 3 | 21 | FANVHKVFGASNVSKMLQEVP |
| 4 | 29 | ALQQQVMELQAELAYAKAELAHYKLQQAN |
| 5 | 11 | IFAPYFPPDEP |
| 6 | 41 | IIKPCLEWIKCPESQGNATLFLAKFYGRIGLLNLITNATEH |
| 7 | 41 | GPTSSETVNAEWHPPPKTYDIRHVAKDTNVDIKGKTQFKRV |
| 8 | 50 | LLKNTNMEIGETSSRVQTEKINEAVENQVNLELTLGFDCQSTKGKKILDK |
| 9 | 15 | TGNWHLCQAAVETVL |
| 10 | 15 | RPCLQWIETPEAQGH |
| 11 | 28 | MYGGMIKKSMEELDHVKWLLAYCKQNNH |
| 12 | 21 | SSQSFGRHETVDDFIQIPYIF |
| 13 | 21 | GKILKPKPRVGSVDSATMLKS |
| 14 | 23 | DILHSHVSLPPFPEFSTCDDFND |
| 15 | 25 | RRPGSPSMNSEESVTTTACLETGIG |
